# Surgical External Iliac Artery Access for Transcatheter Aortic Valve Replacement Is a Safe, Suitable Alternative to Common Femoral Artery Access

**DOI:** 10.7759/cureus.40028

**Published:** 2023-06-06

**Authors:** Justin M George, Christopher M Hatzis, Lukas Ritzer, Sahil Khera, Gilbert Tang, Annapoorna Kini, Peter Faries, Rami O Tadros

**Affiliations:** 1 Vascular Surgery, Icahn School of Medicine at Mount Sinai, New York, USA; 2 Interventional Cardiology, Icahn School of Medicine at Mount Sinai, New York, USA; 3 Cardiovascular Surgery, Icahn School of Medicine at Mount Sinai, New York, USA; 4 Cardiology, Mount Sinai Hospital, New York, USA

**Keywords:** transcatheter aortic valve repair, femoral artery access, external iliac artery access, peripheral artery disease, tavr'

## Abstract

Background

Many patients undergoing transcatheter aortic valve replacement (TAVR) have peripheral artery disease necessitating surgical access. This study reviews the preoperative risk factors, procedural characteristics, and outcomes in patients undergoing surgical common femoral artery (CFA) and external iliac artery (EIA) access through a retro-inguinal groin incision for TAVR.

Methods

A single-center TAVR database was retrospectively analyzed for patients undergoing surgical cutdown (January 1, 2016 - December 31, 2020). Access sites were evaluated on preoperative imaging. Data on demographics, imaging, procedural characteristics, and outcomes were collected. The vascular surgeon selected the cutdown site.

Results

A hundred and thirty TAVR patients had surgical cutdown. The choice of access site was either the common femoral artery (82 patients, 63%) or the iliac artery (48 patients, 37%). There was no difference in age, BMI, or medical risk factors. There was no difference in iliac diameter or circumferential iliac calcium. In the iliac group, there was a smaller mean CFA size and a higher incidence of circumferential CFA calcium. In the femoral group, there was: a lower mean sheath-to-CFA ratio, a trend toward increased unplanned endarterectomy, and a higher incidence of 30-day readmission. There was no difference in adjunct procedure use.

Conclusion

EIA surgical access had similar complication rates and length of stay with a reduced tendency for unplanned endarterectomy when compared to CFA access. The EIA is a suitable access site for TAVR in select patients.

## Introduction

Many patients undergoing transcatheter aortic valve replacement (TAVR) have concomitant peripheral arterial disease (PAD) and require surgical access for delivery of the endoprosthesis [1-2]. Surgical femoral cutdown avoids vascular complications in select patients and anatomy; however, the common femoral artery may be inaccessible or require endarterectomy due to severe atherosclerotic disease [3].

Early in the TAVR experience, the only alternative access was transapical access which was associated with significantly increased morbidity and complication rates [4]. Since this initial experience, data has been published regarding additional access sites including transaxillary, transcarotid, transcaval, and direct aortic access [5-9]. While less morbid than transapical access, these alternative access sites all have their own associated morbidities and procedural complication rates.

The external iliac artery is an alternative surgical access site in patients with severe common femoral disease. There are small series describing technical success using retroperitoneal surgical exposure [10]. These cases were done through traditional transabdominal exposure. There is a paucity of data for outcomes of retro inguinal surgical external iliac artery exposure for TAVR access in patients with severe common femoral artery disease.

The objective of this study is to review preoperative medical and radiographic risk factors, procedural characteristics, and postoperative outcomes in patients undergoing open surgical common femoral artery and open surgical external iliac artery access through a retro inguinal groin incision for TAVR.

## Materials and methods

Data source

This was a single-center, retrospective analysis of patients who underwent open surgical access (‘cutdown’) during TAVR procedures from January 1, 2016 - December 31, 2020. All cases took place at a single, tertiary care hospital. Procedural teams included faculty from vascular surgery, cardiology, and cardiothoracic surgery. All patient data were collected prospectively, extracted from the electronic medical record, and entered into a secure research database. This study was approved by the Institutional Review Board at Mount Sinai Hospital (IRB approval #2101566) and permission was given to use this data without the need for informed consent, given the retrospective and de-identified nature of the data.

Patient cohort & variable definitions

We reviewed all TAVR cases at Mount Sinai Hospital from 2016-2020 and identified all patients who underwent TAVR via open surgical access (n=130). We then divided cases into two cohorts based on the access level: common femoral artery or external iliac artery. All data variables were divided into four distinct categories - demographic data, vascular anatomy, operative characteristics, and postoperative outcomes. Demographic variables included general variables such as age, gender, race, and body mass index, as well as vascular-specific variables including vascular comorbidities (diabetes mellitus, end-stage renal disease, peripheral artery disease, and tobacco use). Prior to intervention, all patients had CT imaging obtained in order to further characterize the vascular anatomy for procedural planning. Anatomical variables of interest included femoral artery diameter, extent of femoral artery calcification, iliac artery diameter, and extent of iliac artery calcification. All CT images were interpreted and corroborated by two vascular surgeons blinded to the surgical intervention received. Operative characteristics analyzed included urgency of procedure (elective vs. non), procedure completion, successful device implementation, the ratio of sheath: femoral artery diameter, type of anesthesia administered, and adjuncts used during index procedure (angioplasty, stenting, lithotripsy, unplanned endarterectomy). Both perioperative and long-term (30-day) postoperative outcomes were reviewed. Perioperative outcomes of interest included major adverse limb events (MALE), stroke, hemoglobin drop > 3 points, transfusion requirement, hematoma, and lymph leak. Long-term operative outcomes included 30-day readmission and reoperation rates, 30-day mortality rate, length of hospital stay, and disposition upon hospital discharge.

Procedure

**Figure 1:**
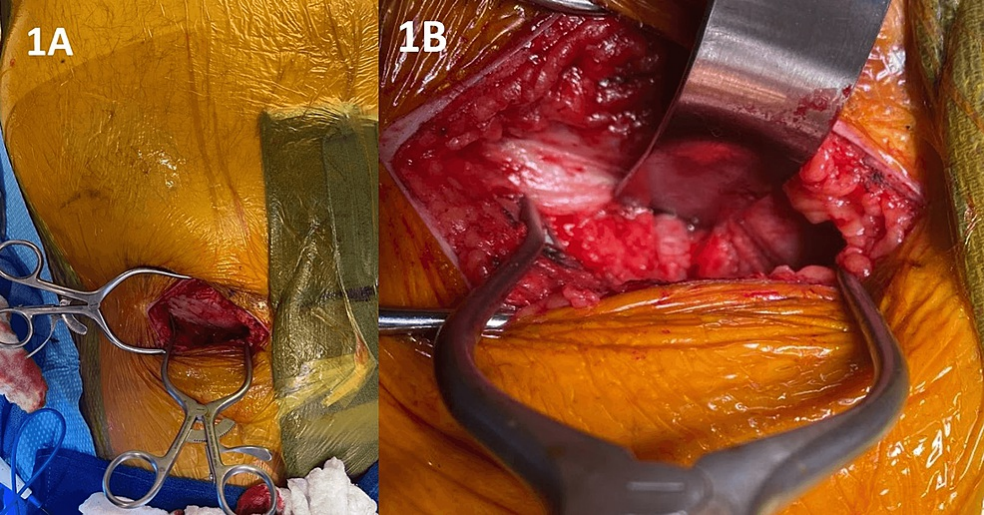
Example of Transverse Surgical Incision Made for External Iliac Artery Exposure and Access for TAVR The inguinal ligament is exposed at the superior aspect of the incision. Retraction of the inguinal ligament allows visualization of the external iliac artery.

**Figure 2:**
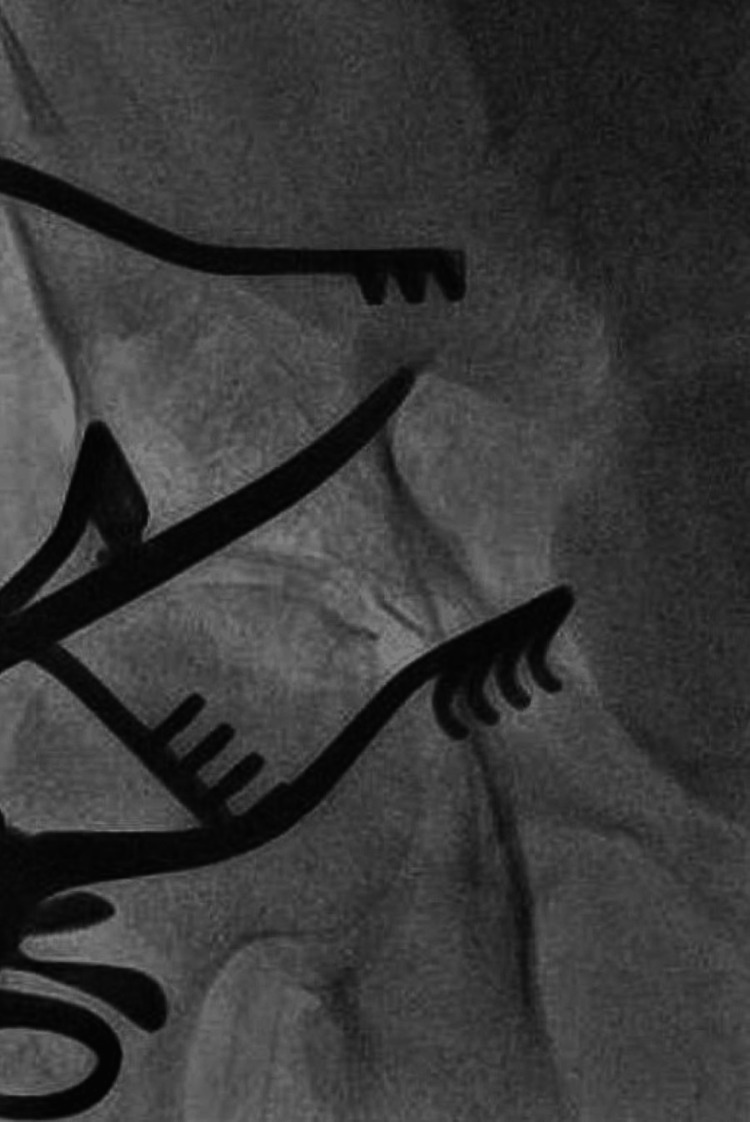
Fluoroscopic Demonstration of Proposed Arterial Access Site Metzenbaum scissor tip (Aesculap, Germany) used as a marker. Scissor placed within the surgical incision, between Weitlaner retractor blades (Ruggles-Redmond, Germany), with the tip placed on the anterior surface of the external iliac artery at the proposed arterial puncture site.

**Figure 3:**
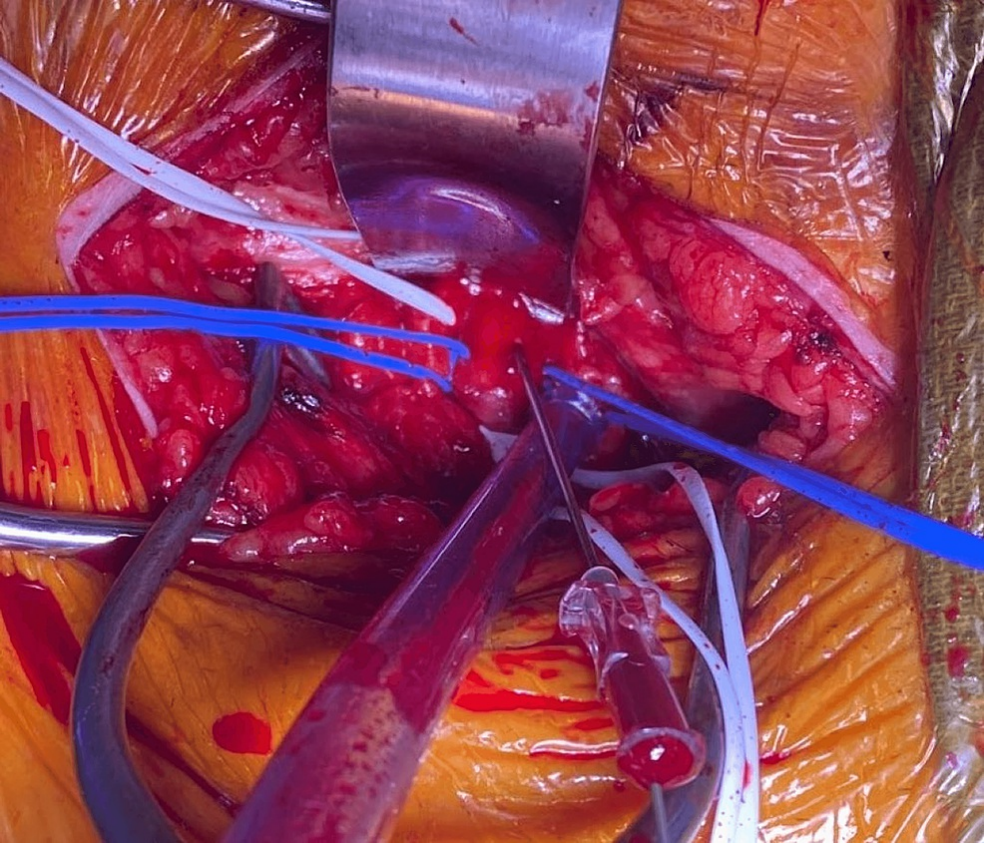
Direct Access of External Iliac Artery for Eventual Placement of TAVR Sheath External iliac artery (white vessel loops) and inferior epigastric arteries (blue vessel loops) have been circumferentially dissected and controlled. Access to the external iliac artery was done under direct visualization with an 18-gauge access needle.

**Figure 4:**
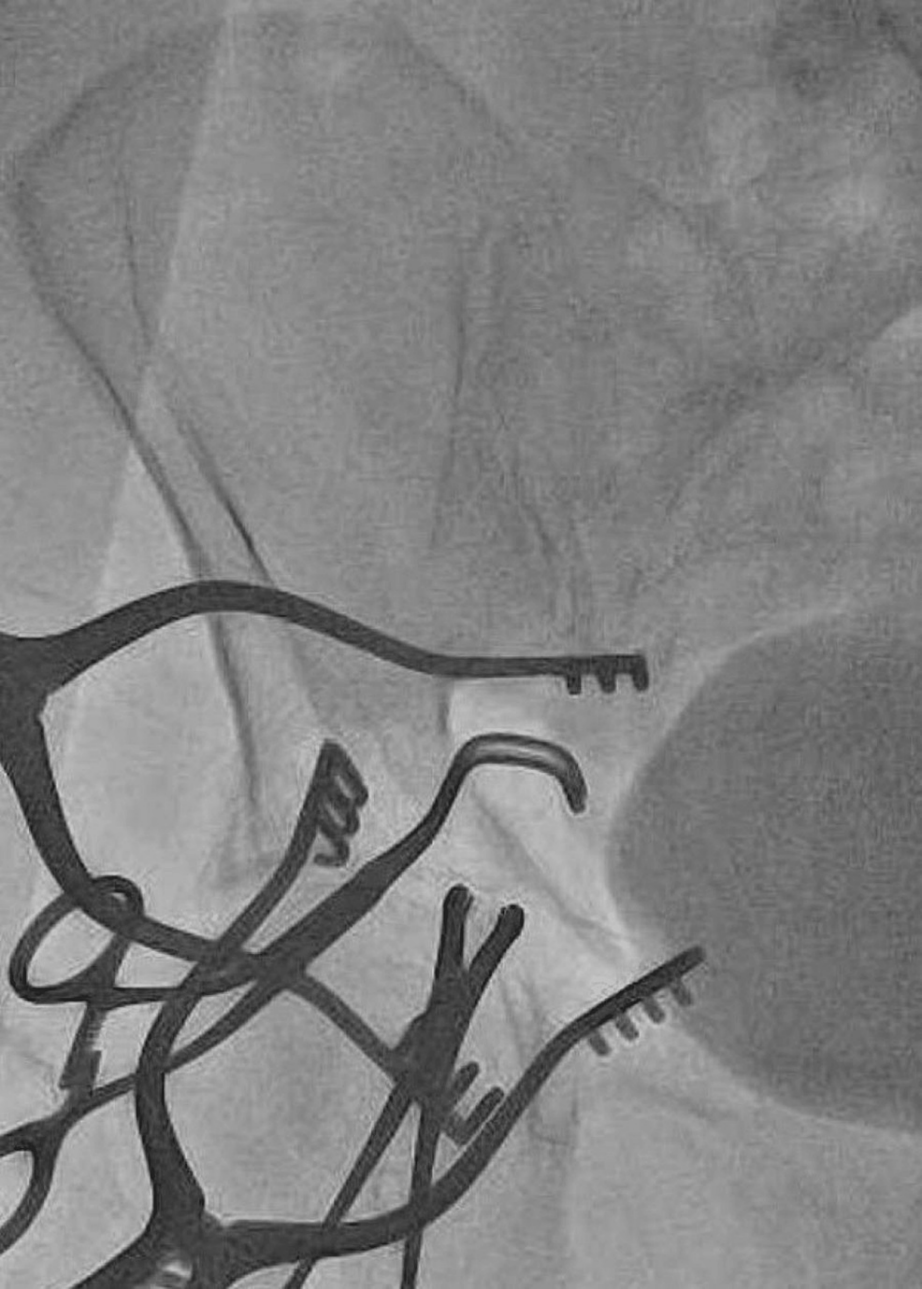
Fluoroscopic Demonstration of Level of Angled Vascular Clamps Providing Proximal and Distal Control during Repair of External Iliac Arteriotomy Weitlaner retractor blades were placed within the surgical incision, exposing the external iliac artery. Two angled vascular clamps (Jarit, Germany) are placed within the surgical incision, providing proximal and distal control of the external iliac artery. Exposure is sufficient for direct repair of arteriotomy with 6-0 interrupted Prolene suture.

All procedures began with the obtainment of retro inguinal exposure. A transverse incision is made in the skin at the level of the inguinal ligament and the ligament is mobilized along its entire length (Figures 1A, 1B). The inguinal ligament is then retracted cephalad to gain access to the retroperitoneum and the external iliac artery is followed proximally until a soft portion is encountered and circumferentially controlled. With sufficient mobilization and retraction of the inguinal ligament, access up to the level of the common iliac artery can be easily achieved (Figures 2, 3, 4).

## Results

Patients

Between January 2016 and December 2020, a total of more than 1,800 TAVR cases were performed at Mount Sinai Hospital. Of these procedures, 130 utilized a surgical cutdown to obtain arterial access for large-bore sheath placement. Cases were divided into two cohorts, those cases where femoral access was obtained (n=82) and those where iliac access was obtained (n = 48). There were no significant differences in background demographic data between the group receiving femoral access as opposed to iliac access (Table 1). Both cohorts had high levels of comorbid hypertension, congestive heart failure, coronary artery disease, peripheral artery disease, and tobacco use. The majority of patients in both cohorts (femoral 71%; iliac 65%) had prior percutaneous femoral access. Prior femoral cutdown was rare (< 20%) in both cohorts.

**Table 1:** Demographic Data in Patients Undergoing Femoral Cutdown and External Iliac Cutdown for TAVR Access COPD: chronic obstructive pulmonary disease

	Femoral	Iliac	
	N = 82	N = 48	p value
Gender, Male %	55	50	0.72
Race, %			
White	74	69	0.55
Black	6	10	0.50
Other	20	21	1.00
Age, years (SD)	81.1 (8.5)	80.0 (8.0)	0.38
Body Mass Index (SD)	26.7 (5.2)	28.0 (6.0)	0.20
Comorbidities, %			
Hypertension	96	98	1.00
Diabetes Mellitus	40	50	0.36
Congestive Heart Failure	52	58	0.59
COPD	27	35	0.33
Coronary Artery Disease	89	96	0.21
End-Stage Renal Disease	11	10	1.00
Peripheral Artery Disease	67	75	0.43
Current/Prior Tobacco Use	73	81	0.39
Prior Femoral Cutdown, %	12	17	0.60
Prior Femoral Percutaneous Access, %	71	65	0.56

Anatomy

Anatomic data was gleaned from preoperative CT imaging. Femoral artery diameter measurements were obtained. Femoral artery diameter was significantly smaller (p < 0.001) in cases where iliac artery access was obtained (5.1 +/- 1.4mm) as opposed to cases where femoral artery access was obtained (7.0 +/- 2.1mm). While there was no difference in the extent of iliac artery calcification, iliac artery access cases did have a significantly higher rate (p<0.001) of circumferential femoral artery calcification. There was no difference in iliac artery diameter between the two cohorts (Table 2).

**Table 2:** Vascular Anatomy in Patients Undergoing Femoral Cutdown and External Iliac Cutdown for TAVR Access

	Femoral	Iliac	
	N = 82	N = 48	p value
Diameter Femoral Artery, mm (SD)	7.0 (2.1)	5.1 (1.4)	< .001>
Femoral Artery Calcification Extent, %			
Circumferential, %	17	50	< .001>
Anterior, %	3	0	0.53
Posterior, %	54	40	0.15
Iliac Artery Diameter, mm (SD)	7.8 (1.8)	7.9 (1.5)	0.89
Iliac Artery Calcification Extent, %			
Circumferential, %	16	13	0.80
Anterior, %	16	13	0.80
Posterior, %	12	10	1.00

Outcomes

The majority of cases in both cohorts were elective and completed successfully with the implantation of a transcatheter valve. The sheath-to-femoral artery ratio was calculated for both groups with a significantly lower ratio (p < 0.001) in the femoral artery access cohort. The sheath-to-femoral artery access ratio was 0.91 + /-0.34 for the femoral access cohort, however, the ratio was > 1 (1.2 + /-0.44) in the iliac artery access cohort. There was variable use of adjunct interventions during the index procedure. Angioplasty of the external and/or common iliac arteries was the most commonly utilized adjunct in both cohorts (11% and 19%, respectively). There were no significant differences in unplanned endarterectomy, however, there was a trend (p = 0.06) towards higher occurrence in the femoral access cohort (19%) as compared to the iliac access cohort (6%) (Table 3). The most common perioperative complications for both cohorts were hemoglobin drop of more than 3 grams/L and intraoperative or postoperative transfusion. More severe complications such as MALE, stroke, and hematoma formation were rare for both cohorts. There was a higher incidence (p = 0.05) of 30-day readmission in the femoral artery access cohort (12%) as opposed to the iliac artery access cohort (2%). There were no differences in 30-day reoperation or mortality rates. The disposition on discharge was similar for both cohorts with ~75% of each cohort being discharged to home and ~25% of each cohort being discharged to subacute rehab (Table 4).

**Table 3:** Operative Characteristics in Patients Undergoing Femoral Cutdown and External Iliac Cutdown for TAVR Access

	Femoral	Iliac	
	N = 82	N = 48	p value
Elective Procedure, %	99	100	1.00
Procedure Completed, %	100	100	1.00
Successful Device Implementation, %	100	100	1.00
Sheath:Femoral Artery Ratio	0.91 (0.34)	1.2 (0.44)	<0.001
General Anesthesia, %	100	100	1.00
Adjuncts During Index Procedure, %			
Angioplasty of Iliac Artery	11	19	0.29
Stenting of Iliac Artery	6	6	1.00
Lithotripsy of Iliac Artery	0	5	0.13
Unplanned Endarterectomy	19	6	0.06

**Table 4:** Postoperative Outcomes in Patients Undergoing Femoral Cutdown and External Iliac Cutdown for TAVR Access

	Femoral	Iliac	
	N = 82	N = 48	p value
Rates of Perioperative Complications, %			
Hemoglobin Drop >3, %	18	15	0.64
Intraoperative Transfusion, %	12	19	0.32
Postoperative Transfusion, %	11	17	0.42
Postoperative Hematoma, %	5	6	0.71
Postoperative Lymph Leak, %	0	0	1.00
Postoperative Stroke, %	5	8	0.47
Major Adverse Limb Events, %	2	2	1.00
30-Day Reoperative Rate, %	2	0	0.53
30-Day Readmission Rate, %	12	2	0.05
30-Day Mortality Rate, %	5	4	1.00
Length of Stay, days (SD)	4.4 (3.8)	4.4 (4.0)	0.93
Disposition, %			
Home	76	77	1.00
Subacute Rehab	21	21	1.00
Death During Index Hospital Stay	4	2	1.00

## Discussion

The risk factors for PAD, including diabetes, hyperlipidemia, smoking, and chronic renal insufficiency, overlap with those of aortic stenosis [11,12]. PAD, thus, is a frequent comorbidity encountered in patients undergoing TAVR ranging from 27.8% in the Partner B Trial to 41.3% in the U.S. CoreValve Trial [1,2,13]. This can lead to significant issues with large bore arterial access for TAVR.

The transfemoral approach is typically the first choice for access because of its relatively low-risk profile, proceduralist comfort, and ability of the femoral artery to tolerate large sheath sizes [14]. Vascular complication rates for early-generation transfemoral TAVR have been reported between eight and 30.7% and are a significant cause of postoperative morbidity and mortality [2,15]. Even with lower profile newer generation devices, major access site complications can range from 6 to 20%, and the common femoral artery is deemed unsuitable requiring alternative access sites in up to 15% of cases [16,17].

Several risk factors have been associated with increased risk for access site complications including female sex, sheath to femoral artery ratio, peripheral arterial disease, and femoral artery calcification [18,19]. These factors are routinely assessed when reviewing access sites in TAVR patients. In this cohort, open surgical access was exclusively chosen in cases where extensive calcification in the femoral vessels would have otherwise led to a compromised or unsafe percutaneous arteriotomy closure. When the decision to proceed with surgical access is made, these characteristics are also considered when determining if common femoral access is acceptable or if external iliac access is required. The external iliac artery was selected more frequently in this series in patients with smaller femoral arteries, higher sheath-to-femoral artery ratio, and circumferential femoral calcium.

The external iliac artery is typically larger with less calcific disease than the common femoral artery. Typically, the external iliac artery is accessed through a retroperitoneal approach. This involves the division of abdominal wall musculature and fascia and is a more invasive exposure that may be associated with more morbidity and longer postoperative recovery. Retroinguinal exposure through an oblique groin incision, the exposure of choice in this cohort, is less invasive and has been well described [20]. Using this approach, patients recovered well with similar lengths of stay and complication rates as those who had common femoral artery exposure. Furthermore, accessing the external iliac artery in this manner leads to similar disposition rates to home.

There are several limitations to this study. It is retrospective and non-randomized. The decision for external iliac vs common femoral exposure was made based on axial imaging and assessment of risk factors by a single vascular surgeon and thus subject to bias. Nevertheless, the purpose of this study was to demonstrate the feasibility of retro inguinal large bore external iliac arterial access for TAVR.

There will be more TAVR procedures performed as it is associated with significantly lower rates of composite death, stroke, or rehospitalization when compared to open surgical repair in patients deemed low surgical risk [21]. Access site assessment and selection by the Heart Team, including vascular surgeons, will continue to be crucial [22]. This series demonstrates the safety and feasibility of retro inguinal external iliac artery exposures.

## Conclusions

External iliac artery surgical access can be safely achieved with no additional morbidity or mortality when compared to traditional surgical common femoral artery exposure. External iliac artery access through a retro inguinal groin approach is associated with decreased rates of unanticipated endarterectomy with no increased complication rate. The external iliac artery is an appropriate access site for large-bore arterial access in select patients such as those with smaller femoral arteries, high sheath-to-femoral artery ratios, and circumferential femoral artery calcium.
